# Knowledge about Chagas disease among Primary Health Care professionals in a municipality located in northeastern Brazil

**DOI:** 10.1371/journal.pntd.0014000

**Published:** 2026-02-09

**Authors:** Marcio Cerqueira Almeida, Jorgana Fernanda Souza Soares, Ronnei Silva Santos, Gilmar Ribeiro, Renato Barbosa Reis, João Marcos Bastos Araújo, Lidiany Menezes Barbosa, Tarcísio Oliveira Silva, Cícera Nunes Souza, Kelle Karolina Ariane Ferreira Alves, Fernanda Cardoso Lanza, Paulo Cainam Guimarães do Nascimento, Claudilson José Carvalho Bastos, Roque Aras Júnior, Isabel Cristina Britto Guimarães, Luciano Kalabric Silva, Bruno Solano de Freitas, José Luiz Moreno Neto, Mitermayer Galvão Reis

**Affiliations:** 1 Oswaldo Cruz Foundation, Gonçalo Moniz Institute, Salvador, Bahia, Brazil; 2 Irecê Faculty, Irecê, Bahia, Brazil; 3 Faculty of Medicine of Bahia, Federal University of Bahia, Salvador, Bahia, Brazil; 4 Medical Residency Program in Radiology at Obras Sociais Irmã Dulce, Bahia State Health Department, Salvador, Bahia, Brazil; 5 Salvador University, Salvador, Bahia, Brazil; 6 Bahia State Health Department, Salvador, Bahia, Brazil; 7 Health Department of the municipality of Irecê, Irecê, Bahia, Brazil; 8 University of Bahia State, Salvador, Bahia, Brazil; 9 Ana Nery Hospital, Salvador, Bahia, Brazil; 10 Yale University, School of Public Health, Department of Epidemiology of Microbial Diseases, New Haven, Connecticut, United States of America; International Atomic Energy Agency, AUSTRIA

## Abstract

Knowledge about Chagas disease (CD) among health professionals is essential to control this public health problem. The objective was to evaluate the knowledge about CD among these professionals. A descriptive cross-sectional study was conducted between April and September 2023, in the city of Irecê, Bahia State, Brazil. Data were collected using a standardized questionnaire and analyzed descriptively. Of the 257 participants, 226 (87.9%) claimed to know the etiological agent, although only 173 (76.5%) recognized it as a protozoan. Regarding the modes of transmission, all workers recognized the vector-borne route, but only 102 (39.7%) identified the vertical route. The majority of workers identified the heart as the affected organ (n = 255; 99.2%). The most identified signs/symptoms in the acute phase were fever (n = 196; 76.6%) and edema (n = 218; 85.2%); in chronic cases, it was recognized that they can be asymptomatic, but the majority recognized that electrocardiographic changes and congestive heart failure may be present. Regarding etiological treatment, 175 (72.0%) acknowledged its existence, but 122 (65.9%) could not state the recommended medication; and for 189 (73.5%), CD is incurable. Regarding the vector insect, 210 (82.0%) reported knowing it. Concerning the service to which located triatomine bugs should be sent, 165 (65.7%) identified the Zoonoses Control Center, and that the precaution to be taken when handling triatomine bugs was to protect their hands; for 234 (91.8%) of the participants, the procedure in case of a triatomine bite in humans was to perform serological tests, and 243 (94.6%) had never had access to information about CD. The health workers’ knowledge about CD was incipient and differed among occupational categories. For accurate surveillance of CD, training should be offered to health professionals, covering everything from signs/symptoms to the investigation of household and entomological contacts.

## Author summary

Chagas disease (CD) is a neglected disease that affects millions of people in several countries around the world. In Brazil, it represents a public health problem, but not all cases are diagnosed. The lack of knowledge about the disease among primary health care (PHC) professionals represents a significant barrier to timely diagnosis and effective treatment, which can result in the worsening of the clinical condition of affected individuals and the spread of the disease. The knowledge of primary care health professionals was evaluated in a northeastern Brazilian municipality classified as a risk area for vector-borne transmission of *T. cruzi*. In the studied municipality, the knowledge among PHC professionals about CD was limited and unequal among occupational categories, which may compromise the effectiveness of surveillance actions, from initial clinical suspicion to the adoption of prevention and control measures. This reality may reflect the situation in other endemic areas of the disease. Our results indicate an urgent need for continuing education for workers regarding CD public programs and policies, in order to improve surveillance in regions at risk for the disease, as well as to provide adequate care to its carriers.

## Introduction

Chagas disease (CD), also known as American trypanosomiasis, is a neglected tropical disease whose etiological agent is the protozoan *Trypanosoma cruzi*, belonging to the class Kinetoplastea and the family Trypanosomatidae [1]. Described in 1909 by the Brazilian physician and researcher Carlos Chagas, CD continues to be a serious public health problem in Latin America and, more recently, has become a focus of attention in previously non-endemic countries due to cases of congenital transmission, as women from endemic areas are not adequately screened during prenatal care [2–4].

In Latin America alone, it is estimated that 75 million people are at risk of infection, 30,000 new cases occur annually, with 12,000 deaths, and approximately 9,000 newborns are infected during pregnancy [2]. In Brazil, in 2019, it was estimated that there were 2,164,570 people infected with T. *cruzi* [5].

Due to its high direct and indirect social cost, the interruption of CD transmission via vector, oral, blood transfusion, organ transplant, and congenital routes by 2030 was included in the global plan of the United Nations (UN) and the World Health Organization (WHO) [6]. To achieve these goals, major political and administrative challenges urgently need to be overcome so as not to risk losing all the progress achieved [7].

In Brazil, the progress made refers mainly to the eradication of *T. cruzi* transmission by *Triatoma infestans*, and the compulsory notification of chronic CD cases [8]. For these advances to be sustainable and for others to be achieved, it is important that the surveillance system be accurate, which includes delineating the risk for the disease and implementing prevention and control measures. In this sense, primary health care (PHC) is essential as it is the 1^st^ level of care in the Health Care Network (RAS) of the Unified Health System (SUS), Brazil’s universal public health system.

Considered the preferential entry point for users into the SUS and the coordinator of care within the RAS, PHC needs to have high resolution in health promotion and disease prevention actions, which includes the ability to diagnose and treat the most prevalent conditions in the territories covered by multidisciplinary primary care teams, among which the family health teams (ESF) stand out. Each ESF is responsible for the care of 2,000–3,500 people and is composed, at a minimum, of a physician, nurse, nursing assistant/technician, and community health agents, and may include other professionals such as endemic disease control agents and those from the oral health teams – dentist and dental assistant/technician [9].

Therefore, the knowledge of professionals working in PHC in Brazil, regarding the forms of transmission, diagnostic methods, and treatment, is essential so they can suspect, diagnose, notify, and adequately treat people affected by the disease, and initiate the epidemiological investigation process to identify new cases and areas with the presence of *T. cruzi*-infected triatomine bugs. This is particularly relevant in the municipality of Irecê-Bahia, in Brazil, an area classified as high risk for vector-borne transmission of *T. cruzi* [10].

Despite the importance of health professionals knowledge about CD for the operationalization of surveillance, few studies have been conducted to evaluate it [11], a reality that extends to Brazil. A study conducted with community health agents working in PHC in a regional health superintendency of the state of Minas Gerais-Brazil, identified limited knowledge about CD among these professionals, a lack of training offered on the topic, and the absence of an established health care network for disease assistance [12].

Furthermore, a study conducted with physicians, nurses, nursing assistants, and community health agents from PHC in two municipalities of Paraná between September 2004 and July 2005, found that the majority of health professionals were unaware of the modes of CD transmission. Specifically among physicians, who are responsible for the diagnostic confirmation of CD, the majority identified the Machado-Guerreiro test as the exam to be requested for diagnosis, an obsolete technique for this purpose [13]. Existing studies in Brazil have primarily involved community health agents and endemic disease control agents, who are tasked with conducting family follow-ups via home visits and identifying risk factors for zoonoses within localities, respectively. These investigations [12,14] have identified that the awareness and understanding of CD among these professionals are often superficial. This finding underscores the necessity of implementing comprehensive capacity-building initiatives to enhance the effective of preventive actions at the community level.

Based on the foregoing, and considering the “epidemiological silence” in Latin America—where fewer than 10% of individuals are diagnosed and only about 1% receive antiparasitic treatment for CD, largely due to limited knowledge among health professionals regarding its clinical management [14]—the urgency of addressing this gap is evident. It is therefore essential that PHC professionals understand the specific characteristics of the disease to strengthen health surveillance, provide appropriate care for affected individuals, and promote community health education for disease prevention.

Thus, this study aimed to describe the knowledge about CD among professionals working in the PHC of the municipality of Irecê - Bahia in Brazil.

## Methods

### Ethical statement

In accordance with Resolution No. 466/2012 [15], the study was approved by the Research Ethics Committee of the Instituto Gonçalo Moniz – CEP IGM (Opinion No. 38574820.0.0000.0040). Prior to data collection, all participants signed two copies of the informed consent form (ICF) (S1 Appendix) - one remaining with the participant and the other with the researcher. The ICF datailed the study objectives, potential risks and benefits of participating in the study, the assurances of confidentiality and anonymity, and the participant’s right to withdraw from the study at any stage.

### Study design, population and area

**Fig 1:**
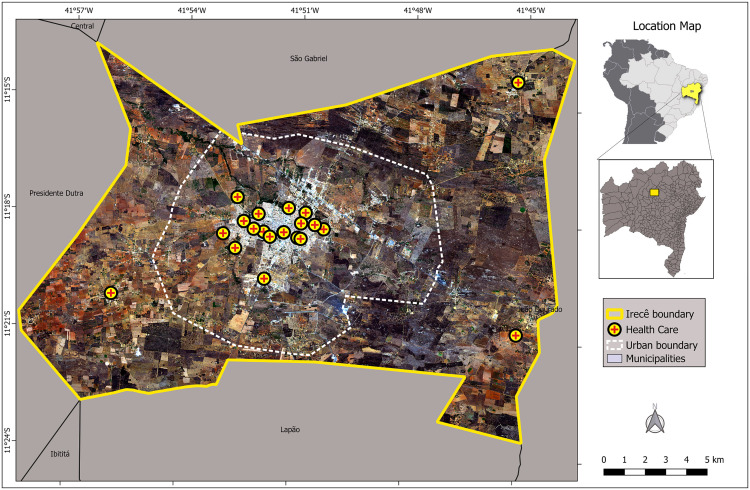
Map of Irecê, Bahia, Brazil, showing the spatial distribution of health care units. Delimitation of the municipality of Irecê (yellow line) and location map of Bahia and Brazil (https://www.ibge.gov.br/geociencias/organizacao-do-territorio/malhas-territoriais/15774-malhas.html). Urban limit of Irecê (white dashed line https://irece.ba.gov.br/servicos/secretaria_de_infraestrutura__e_servicos_publicos_-5/mapas_e_dados-25). [16] https://doi.org/10.6084/m9.figshare.29538869.

A cross-sectional, descriptive, census study, whose population comprised the 324 professionals working in the 22 ESF teams that constitute the PHC Units (UAPS) in Irecê, between the months of April and September 2023 (Fig 1).

### Data collection tool and procedure

The data collection questionnaire (S2 Appendix) was developed by two researchers (MCA and JFSS) based on a literature review and was pilot-tested with 25 health professionals from three UAPS to identify potential issues in question comprehension and to refine the instrument accordingly. The instrument is comprised of four sections: I) sociodemographic characteristics of the worker; II) work-related characteristics; III) knowledge regarding CD; and IV) health surveillance practices for CD. In this study, we present the results corresponding to sections I, II, and III.

Prior to submitting the research project to the CEP IGM, a meeting was held with the Municipal Health Secretariat (SMS) of Irecê to arrange for data collection in the UAPS and to obtain of UAPS names, addresses, and a nominal list of the health professionals working in each unit. The SMS subsequently informed all UAPS of its authorization for the study. In each UAPS, the research and its objectives were explained to the health professionals, inviting them to participate in the study by signing the ICF. The questionnaire was applied primarily in a private setting. Health professionals who were not found at the UAPS after three visits were not included in the study.

### Study variables

The variables for section I included gender (male, female); age group (20–36 years, 37–43 years, 44–51 years, 52–71 years); Race/skin color (Black, Brown/Mixed, White); education level (incomplete primary to incomplete secondary education, complete secondary to incomplete higher education, complete higher education, postgraduate lato or stricto sensu); place of birth (Irecê, another city); city of residence (Irecê, another city); place of residence (neighborhood where you work, another neighborhood).

Section II comprised the following variables: occupation (physician, nurse, nursing technician/assistant, community health agent, dentist, dental office assistant/dental hygiene technician, receptionist, pharmacy assistant, general services, others), with the occupational stratification presented in this paper referring to the basic ESF team – physicians, nurses, nursing assistants/technicians, community health agents; time in the occupation (Less than 2 years, 2–5 years, 6–14 years, 15–19 years, 20–46 years); time working at the UAPS (Less than 1 year, 1–3 years, 4–6 years, 7–14 years, 15–25 years); weekly working hours (Less than 20 hours, between 20 and 40 hours, 40 hours or more); employment bond (formally registered employee, employee without a formal contract, legal entity/self-employed/own account, statutory public servant, other - cooperative member, oral “verbal contract”); previous work in the ESF (yes, no).

The variables for section III concerning worker’s knowledge were: knows the etiological agent (yes, no); etiological agent (virus, bacterium, fungus, protozoan); forms of transmission (vector-borne, blood transfusion, accidents with blood/biological material, vertical, oral); main organs affected (heart, spleen, large intestine, brain, esophagus, kidney, liver); clinical manifestations of the acute phase (fever, adenomegaly, diarrhea, edema, emesis, splenomegaly, hepatomegaly, persistent cough, Romaña’s sign, chagoma of inoculation); clinical manifestations of the chronic phase (asymptomatic, electrocardiographic changes, megacolon, megaesophagus, congestive heart failure, thromboembolic phenomena, and dyspnea); existence of etiological treatment (yes, no), medication for etiological treatment (benznidazole, nifurtimox, amiodarone, propranolol), existence of a cure for CD (yes, no); knows the “barbeiro” (triatomine bug) (yes, no); species to be identified as “barbeiro” (*Nezara viridula*, *Triatoma lenti*, *Triatoma infestans*, *Cosmoclopius nigroannulatus*, 4^th^ nymph stage of *Rhodnius* sp), service for forwarding a triatomine bug (Triatomine Identification Post – PIT, Zoonoses Control Center – CCZ, Municipal Health Secretariat - SMS); care when handling a triatomine bug (use gloves or protect hands with a plastic bag, the “barbeiro” should not be handled under any circumstances; don’t know); guidance to be given in case of a triatomine bite in humans (perform blood tests to monitor serology, start treatment for the disease immediately, wait for symptoms to appear, don’t know); personal access to information about CD (during health-related training course, training course, informally in daily work, cases in the family, basic education - Primary and Secondary School, never had access to information about CD, other source). (S2 Appendix).

For participants who reported familiarity with the insect, triatomine bug identification was confirmed using an illustrated card (S3 Appendix), and their responses were recorded electronically. (S3 Appendix)

### Data processing and analysis

Data were collected and stored in the Research Electronic Data Capture (REDCap). For data processing and analysis, the Statistical Package for the Social Sciences (SPSS) version 21.0 was used. Data were analyzed descriptively using absolute and relative frequencies. As this was a census study, inferential statistical techniques were not used. The complete dataset underlying the presented analyses is available in the Supplementary Information (S4 Appendix).

### Data quality control

Data collection was performed by a previously trained interviewer. The construct validity of the instrument was reviewed by a researcher experienced in CD.

## Results

Of the total of 324 health professionals working in the 22 UAPS, the 25 participants from the pilot study were excluded from the analysis because the questionnaire was modified after their interviews were conducted. Thus, 299 workers were considered eligible for the study, among these, 28 were considered losses and 14 refused to participate, resulting in a final sample of 257 participants (response rate of 86.0%).

Of the 257 participants, the majority identified as female (n = 221; 86.0%); in the age group of 44–51 years (n = 68; 26.5%) and 20–36 years (n = 67; 26.1%); of Brown/Mixed skin color (n = 163; 63.4%); with an education level of Complete Secondary to Incomplete Higher Education (n = 178; 69.3%); born in Irecê (n = 143; 55.6%), residents of the same city (n = 247; 96.1%) and in the same neighborhood where they work (n = 139; 54.1%). Regarding work characteristics, most frequently they were community health agents (n = 108; 42.0%); had been working in the occupation for between 06 and 14 years (n = 73; 28.4%) or between 20 and 46 years (n = 72; 28.0%) and at the UAPS for between 07 and 14 years (n = 67; 26.1%); working a weekly schedule of 40 hours or more (n = 242; 94.2%); were statutory public servants (n = 129; 50.2%); and had not previously worked in the ESF (n = 181; 70.7%). (Table 1).

**Table 1:** Sociodemographic and work characteristics of Primary Health Care (PHC) professionals, Irecê – Bahia, Brazil, 2023.

Variable	n	%
Gender (N = 257)		
Female	221	86.0
Male	36	14.0
Age group (N = 257)		
20 to 36 years	67	26.1
37 to 43 years	62	24.1
44 to 51 years	68	26.5
52 to 71 years	60	23.3
Skin color (N = 257)		
Black	51	19.8
Brown/Mixed	163	63.4
White	43	16.7
Education level (N = 257)		
Incomplete primary to incomplete secondary education	10	3.9
Complete secondary to incomplete higher education	178	69.3
Complete higher education	46	17.9
Postgraduate (*lato or stricto sensu*)	23	8.9
Place of birth (N = 257)		
Irecê	143	55.6
Another city	114	44.4
City of residence (N = 257)		
Irecê	247	96.1
Other	10	3.9
Place of residence (N = 257)		
Neighborhood where they work	139	54.1
Another neighborhood	118	45.9
Occupation (N = 257)		
Physician	15	5.8
Nurse	19	7.4
Nursing Technician/Assistant	33	12.8
Community health agent	108	42.0
Dentist	13	5.1
Dental office assistant/dental hygiene technician	11	4.3
Receptionist	20	7.8
Pharmacy attendant	15	5.8
General services	16	6.2
Others	07	2.7
Time in the occupation (N = 257)		
Less than 02 years	23	8.9
02 to 05 years	43	16.7
06 to 14 years	73	28.4
15 to 19 years	46	17.9
20 to 46 years	72	28.0
Time working at the UAPS (N = 257)		
Less than 01 year	35	13.6
01 to 03 years	58	22.6
04 to 06 years	38	14.8
07 to 14 years	67	26.1
15 to 25 years	59	23.0
Weekly working hours (N = 257)		
Less than 20 hours	06	2.3
Between 20 and 40 hours	09	3.5
40 hours or more	242	94.2
Employment bond (N = 257)		
Formally registered employee	04	1.6
Employee without a formal contract	39	15.2
Legal entity/self-employed/own account	05	1.9
Statutory public servant	129	50.2
Other (cooperative member, oral “verbal contract”)	80	31.1
Previous work in the ESF (N = 256)		
Yes	75	29.3
No	181	70.7

Tables 2, 3, 4, 5 presents the responses stratified by occupational category of the basic ESF team—physicians, nurses, nursing assistants/technicians, and community health agents—in relation to the total number of participants. Of the total of workers, the majority reported knowing who the etiological agent of CD (n = 226; 87.9%), and the protozoan was recognized as such by 173 (76.5%), it is noteworthy that in all professional categories, higher percentages for the aforementioned responses were also observed (Table 2).

**Table 2:** Knowledge of etiological agent, affected organs, and modes of transmission among health professionals, Irecê – Bahia, Brazil, 2023.

Variable	Total (N = 257) n (%)	Physician (N = 15) n (%)	Nurse (N = 19) n (%)	Nursing Asst./Tech (N = 33) n (%)	Community Health Agent (N = 108) n (%)
Knows the etiological agent	226 (87.9)	15 (100.0)	19 (100.0)	28 (84.8)	94 (87.0)
Identification of the etiological agent¹					
*Virus*	26 (11.5)	0 (0.0)	1 (5.3)	2 (6.1)	13 (12.0)
*Bacteria*	22 (9.7)	1 (6.7)	2 (10.5)	2 (6.1)	10 (9.3)
*Fungus*	5 (2.2)	0 (0.0)	0 (0.0)	0 (0.0)	3 (2.8)
Protozoan	173 (76.5)	14 (93.3)	16 (84.2)	24 (72.7)	68 (63.0)
Organs affected by the disease					
Heart	255 (99.2)	15 (100.0)	19 (100.0)	33 (100.0)	108 (100.0)
Spleen	150 (58.4)	6 (40.0)	17 (89.5)	26 (78.8)	62 (57.4)
Large intestine	102 (39.7)	14 (93.3)	7 (36.8)	16 (48.5)	44 (40.7)
Brain	47 (18.3)	2 (13.3)	6 (31.6)	8 (24.2)	13 (12.0)
Esophagus	90 (35.0)	14 (93.3)	6 (31.6)	14 (42.4)	33 (30.6)
Kidney	81 (31.5)	2 (13.3)	3 (15.8)	14 (42.4)	37 (34.3)
Liver	127 (49.4)	6 (40.0)	11 (57.9)	18 (54.5)	53 (49.1)
Modes of transmission					
Vector-borne	257 (100.0)	15 (100.0)	19 (100.0)	33 (100.0)	108 (100.0)
Blood transfusion	142 (55.3)	13 (86.7)	16 (84.2)	16 (48.5)	56 (51.9)
Accidents with blood/biological material	108 (42.0)	10 (66.7)	9 (47.4)	9 (27.3)	40 (37.0)
Vertical	102 (39.7)	5 (33.3)	12 (63.2)	9 (27.3)	37 (34.3)

**Table 3:** Knowledge of clinical manifestations, treatment, and cure of Chagas disease among health professionals, Irecê – Bahia, Brazil, 2023.

Variable	Total n (%)	Physician (N = 15) n (%)	Nurse (N = 19) n (%)	Nurse Asst./Tech (N = 31) n (%)	Community Health Agent (N = 102) n (%)
Clinical manifestations of the acute phase (N = 256)					
Fever	196 (76.6)	13 (86.7)	19 (100.0)	23 (74.2)	77 (75.5)
Adenomegaly	152 (59.4)	13 (86.7)	15 (78.9)	16 (51.6)	68 (66.7)
Diarrhea	77 (30.1)	5 (33.3)	10 (52.6)	7 (22.6)	32 (31.4)
Edema	218 (85.2)	13 (86.7)	18 (94.7)	25 (80.6)	93 (91.2)
Emesis	96 (37.5)	7 (46.7)	10 (52.6)	10 (32.3)	31 (30.4)
Splenomegaly	174 (68.0)	12 (80.0)	13 (68.4)	25 (80.6)	80 (78.4)
Hepatomegaly	167 (65.2)	10 (66.7)	12 (63.2)	21 (67.7)	77 (75.5)
Persistent cough	65 (25.4)	2 (13.3)	2 (10.5)	11 (35.5)	24 (23.5)
Romaña’s sign	129 (50.4)	11 (73.3)	12 (63.2)	20 (64.5)	45 (44.1)
Chagoma of Inoculation	83 (32.4)	13 (86.7)	12 (63.2)	11 (35.5)	26 (25.5)
Clinical manifestations of the chronic phase (N = 257)					
Asymptomatic	167 (65.0)	13 (86.7)	16 (84.2)	19 (57.6)	72 (66.7)
Electrocardiographic changes	255 (99.2)	15 (100.0)	19 (100.0)	32 (97.0)	108 (100.0)
Megacolon	166 (64.6)	15 (100.0)	14 (73.7)	20 (60.6)	72 (66.7)
Megaesophagus	152 (59.1)	15 (100.0)	16 (84.2)	16 (48.5)	59 (54.6)
Congestive heart failure	255 (99.2)	15 (100.0)	19 (100.0)	32 (97.0)	108 (100.0)
Thromboembolic phenomena	150 (58.4)	9 (60.0)	14 (73.7)	15 (45.5)	59 (54.6)
Respiratory distress	208 (80.9)	13 (86.7)	14 (73.7)	29 (87.9)	88 (81.5)
Knowledge of Treatment and Cure					
Existence of etiological treatment	185 (72.0)	8 (53.3)	12 (63.2)	21 (63.6)	82 (75.9)
Knows the indicated medication (N = 185)	63 (34.1)	5 (62.5)	11 (91.7)	7 (33.3)	19 (23.2)
Name of medication (N = 63)					
Benznidazole	42 (66.7)	4 (80.0)	9 (81.8)	4 (57.1)	10 (52.6)
Nifurtimox	18 (28.6)	2 (40.0)	4 (36.4)	1 (14.3)	4 (21.1)
Amiodarone	20 (31.7)	3 (60.0)	2 (18.2)	3 (43.9)	6 (31.6)
Propranolol	17 (27.0)	0 (0.0)	2 (18.2)	2 (28.6)	8 (42.1)
Existence of a cure for Chagas disease	51 (19.8)	3 (20.0)	5 (26.3)	3 (9.1)	17 (15.7)

**Table 4:** Knowledge and management of the “barbeiro” (triatomine bug) among health professionals, Irecê – Bahia, Brazil, 2023.

Variable	Total (N = 256) n (%)	Physician (N = 15) n (%)	Nurse (N = 19) n (%)	Nursing Asst./Tech (N = 33) n (%)	Community Health Agent (N = 108) n (%)
Knows the “barbeiro” (triatomine bug)	210 (82.0)	15 (100.0)	16 (84.2)	29 (87.9)	89 (83.2)
Species identified as “barbeiro” (N = 210)					
*Nezara viridula*	53 (25.3)	4 (26.7)	3 (18.8)	6 (20.7)	25 (28.1)
*Triatoma lenti*	131 (62.3)	12 (80.0)	9 (56.3)	21 (72.4)	55 (61.8)
*Triatoma infestans*	158 (75.2)	14 (93.3)	12 (75.0)	25 (86.2)	66 (74.2)
*Cosmoclopius nigroannulatus*	59 (28.1)	1 (6.7)	5 (31.3)	7 (24.1)	27 (30.3)
4^th^ nymph stage of *Rhodnius sp.*	14 (6.7)	1 (6.7)	3 (18.8)	0 (0.0)	6 (6.7)
Service for forwarding a triatomine bug (N = 251) ¹					
Triatomine Information Post (PIT)	36 (14.3)	4 (30.8)	4 (21.1)	4 (12.1)	10 (9.3)
Zoonoses Control Center	165 (65.7)	12 (92.3)	16 (84.2)	20 (60.6)	78 (72.2)
Municipal Health Secretariat	100 (39.8)	3 (23.1)	2 (10.5)	13 (39.4)	36 (33.3)
Care when handling the triatomine bug					
Use gloves or protect hands	237 (92.2)	14 (100.0)	18 (94.7)	30 (93.4)	104 (97.2)
The “barbeiro” should not be handled	13 (5.1)	0 (0.0)	1 (5.3)	2 (6.3)	3 (2.8)
Guidance after a triatomine bite (N = 255)¹					
Perform blood tests to monitor serology	234 (91.8)	15 (100.0)	18 (94.7)	32 (97.0)	99 (93.4)
Start treatment for the disease immediately	12 (4.7)	0 (0.0)	0 (0.0)	0 (0.0)	3 (2.8)
Wait for symptoms to appear	7 (2.7)	0 (0.0)	1 (5.3)	1 (3.0)	2 (1.9)
Don’t know	2 (0.8)	0 (0.0)	0 (0.0)	0 (0.0)	2 (1.9)

¹ Variations in sample sizes (N) are due to participants who either refused to answer or provided ‘don’t know’ responses to questions in that section.

**Table 5:** Personal access to information about Chagas disease among health professionals, Irecê – Bahia, Brazil, 2023.

Variable	Total (N = 257) n (%)	Physician (N = 15) n (%)	Nurse (N = 19) n (%)	Nursing Asst./Tech (N = 33) n (%)	Community Health Agent (N = 108) n (%)
During health-related training course	72 (28.0)	15 (100.0)	14 (73.7)	19 (57.6)	11 (10.2)
Training course	57 (22.2)	0 (0.0)	0 (0.0)	4 (12.1)	44 (40.7)
Informally in the daily work routine	77 (30.0)	0 (0.0)	3 (15.8)	9 (27.3)	37 (34.3)
Cases in the family	14 (5.5)	0 (0.0)	0 (0.0)	0 (0.0)	4 (3.7)
Basic education (Primary/Secondary School)	13 (5.1)	0 (0.0)	0 (0.0)	0 (0.0)	2 (1.9)
Never had access to information	14 (5.4)	0 (0.0)	0 (0.0)	0 (0.0)	6 (5.6)
Other source	10 (3.9)	0 (0.0)	2 (10.5)	1 (3.0)	4 (3.7)

Regarding the forms of transmission of CD, vector-borne transmission (n = 257; 100.0%), blood transfusion (n = 142; 55.3%), and oral transmission (n = 159; 61.9%) were identified. Conversely, the least recognized modes of transmission were the vertical route (n = 102; 39.7%) and occupational exposure to blood/biological material (n = 108; 42.0%). It is also noteworthy that 31 were unable to answer or did not provide a response to this question (Table 2). Analysis by occupational category revealed notable differences in the recognition of transmission forms. Specifically, blood transfusion was correctly identified by less than half of the nursing assistants/technicians (48.5%, n = 16). On the other hand, it is noteworthy that accidents with blood/biological material were pointed out as a form of transmission only by physicians (n = 10; 66.7%), and the vertical route by nurses (n = 12; 63.2%) (Table 2).

The main organs compromised by the disease were recognized as the heart (n = 255; 99.2%) and the spleen (n = 150; 58.4%). However, other commonly affected organs were less recognized, including the large intestine (n = 102; 39.7%) and the esophagus (n = 90; 35.0%). Similarly, recognition was low for the liver (n = 127; 49.4%), kidneys (n = 81; 31.5%), and the brain (n = 47; 18.3%). Physicians were the only group to predominantly recognize the large intestine and the esophagus (n = 14; 93.3% for both), although their recognition of the liver was considerably lower (n = 6; 40.0%) (Table 2).

Regarding the clinical manifestations of CD in the acute phase, 257 responded and 01 did not know the answer/ignored the question. The professionals identified the following signs and symptoms of CD: fever (n = 196; 76.6%), adenomegaly (n = 152; 59.4%), edema (n = 218; 85.2%), splenomegaly (n = 174; 68.0%), hepatomegaly (n = 167; 65.2%), Romaña’s sign (n = 129; 50.4%). However, the symptoms diarrhea (n = 77; 30,1%), emesis (n = 96; 37.5%), persistent cough (n = 65; 25,4%), and chagoma of inoculation (n = 83; 32,4%) were not recognized as such. Among the occupational category of nurses, the main recognized symptoms of acute CD were fever (100%), edema (94.7%) and adenomegaly (78.9%). With regard to emesis, the occupational category that most frequently reported this symptom was nurses. A minority of community health agents recognized Romaña’s sign (44.1%, n = 45). Similarly, the chagoma of inoculation was identified by a minority of community health agents (25.5%, n = 26) and nursing assistants/technicians (35.5%, n = 11) (Table 3).

Concerning the chronic phase, it was recognized that it can be asymptomatic (n = 167; 65.0%), and electrocardiographic changes (n = 255; 99.2%), megacolon (n = 166; 64.6%), megaesophagus (n = 152; 59.1%), congestive heart failure (n = 255; 99.2%), thromboembolic phenomena (n = 150; 58.4%), and respiratory distress (n = 208; 80.9%) were also identified as signs/symptoms. When observing the percentages of the responses, it can be said that there was similarity between the occupational groups for the clinical manifestations of the chronic phase, The exception was noted among nursing assistants/technicians, as only a minority of this group identified megaesophagus (48.5%, n = 16) and thromboembolic phenomena (45.5%, n = 15) as signs of the chronic phase of CD (Table 3).

When questioned about etiological treatment, 185 (72.0%) of participants acknowledged its existence, although (n = 21; 8.2%) did not know how to answer/ignored the question, among them (n = 03; 15.8%) nurses and (n = 09; 297 8.3%) community health agents. However, of those who affirmed its existence, only a minority (34.1%, n = 63) could name an indicated medication. Among these 63 participants who named a medication, Benznidazole was identified by 42 (66.7%). It is worth noting that Amiodarone was recognized as an etiological treatment by physicians (n = 03; 60.0%). Regarding the existence of a cure for CD, only a minority of participants (19.8%, n = 51) affirmed that a cure exists (Table 3).

Regarding the knowledge about triatomine bugs among the professionals of the four categories under study, 82.0% (n = 210) reported knowing them. Within these categories, professionals identified as the “barbeiro” (triatomine bugs) *Triatoma lenti* (n = 131; 62.3%), *Triatoma infestans* (n = 158; 75.2%), *Cosmoclopius nigroannulatus* (n = 59; 28.1%). The 4^th^ nymph stage of *Rhodnius* sp was recognized as such by only 14 (6.7%) of the respondents (Table 4).

When asked which service triatomine bugs found in households should be referred to, 165 (65.7%) participants identified the Zoonoses Control Center, a response that predominated across all professional categories. However, the designation of Triatomine Identification Posts (PIT) as the referral location by 4 physicians (30.8%) and 4 nurses (21.1%). The precaution identified by participants when handling triatomine bugs was to use gloves or protect their hands with a plastic bag (n = 237; 92.2%), with similar percentages among the professional categories. The guidance to be given in case of a triatomine bite on humans referred to performing blood tests for serological monitoring (n = 234; 91.8%), with no differences in the response across professional categories (Table 4).

Personal access to information about CD most frequently occurred informally in the daily work routine (n = 77; 30.0%), particularly for community health agents (n = 37; 34.3%). On the other hand, knowledge about the disease was acquired during health-related training courses (n = 72; 28.0%), being the main source of information for physicians (n = 15; 100.0%), nurses (n = 14; 73.7%) and nursing assistants/technicians (n = 19; 57.6%) (Table 5).

## Discussion

The predominance of female workers corroborates data from other research, where this gender is prevalent among health professionals [17]. This may favor patient adherence to health services, since gender-related social constructs often influence care practices. In many contexts, women health professionals are more likely to provide person-centered care, which facilitates interaction and interpersonal communication—important characteristics for the follow-up of people with CD who require comprehensive care [18,19].

Regarding the place of residence, the majority of professionals reside in Irecê and in the same neighborhood as the UAPS where they work, have a 40-hour work week, and are public servants, which contributes to a practice consistent with the local epidemiological profile, strengthening the bond with users and ensuring the longitudinality of care. Despite this, it is noted that the majority of professionals had not previously worked in the ESF, which reflects the health training process in Brazil, which prioritizes teaching about SUS management rather than practice in primary care, and minimizes the training of professionals with autonomy, critical awareness, and the capacity to resolve the health problems that affect the population [20].

Considering the endemicity of CD in Brazil [21] and the state of Bahia being classified as high risk for *T. cruzi* transmission [22], the results presented in this study need to be the subject of intervention by health managers. Although most health professionals recognized that the etiological agent is a protozoan, they more frequently failed to recognize accidental transmission through exposure to blood/biological material and vertical transmission as possible routes. However, the majority of physicians recognized the accidental route whereas nurses more frequently identified vertical transmission.

Accidental transmission can occur through different forms: handling of triatomine bugs; experimental work with *T. cruzi*-infected mammals; handling of different *T. cruzi* culture media; performance of surgical procedures and blood collection on individuals with CD. The associated risk factors are entirely related to occupation and include lack of knowledge about this mode of transmission, and the absence or misuse of personal protective equipment [23].

Vertical transmission of *T*. *cruzi* currently represents a serious public health problem, both in endemic and non-endemic areas [24]. The estimate in the Americas is that 2 million women of childbearing age are infected with the protozoan, resulting in the annual birth of 9,000 infected babies [25]. Transmission occurs from the infected mother to the child during pregnancy or childbirth, which is why health professionals need to screen and treat women of childbearing age [26], as well as request serology during prenatal care in areas at risk for *T*. *cruzi* transmission or when the pregnant woman comes from an area endemic for the disease. In this cases, the physicians and nurses are responsible to investigate the history during prenatal consultations [27].

CD is multisystemic, meaning that *T*. *cruzi* can be found in various organs [28,29]. Although the heart was identified by all health professionals as one of the main organs affected by the disease, the esophagus and the large intestine were recognized as such only by physicians. Contradictorily, megaesophagus and megacolon were distinguished as clinical manifestations of the chronic phase of CD by the majority of workers in their respective occupational categories. The digestive form of CD primarily affects the esophagus and the colon *leading to* dysphagia and constipation, which can evolve to megaesophagus and/or megacolon. These dilations are associated with destruction of cells of the enteric nervous system, mainly in Auerbach’s myenteric plexus, leading to peristaltic paralysis and smooth muscle hypertrophy caused by *T. cruzi* infection in these organs [30].

Therefore, the clinical management of megaesophagus and megacolon should facilitate the passage of food to the stomach and stimulate evacuation in order to avoid complications such as malnutrition, fecal impaction with consequent formation of a fecaloma, stercoral ulcer, and sigmoid volvulus. This consequences debilitate the clinical condition of the person affected by the disease and represent potential clinical/surgical emergencies [27]. Furthermore, particularly in endemic areas where the seroprevalence of anti-*T*. *cruzi* is 76.3% among individuals with megacolon [31], health professionals need to be alert to the investigation of the etiology of megacolon/megaesophagus in order to diagnose and administer antiparasitic medication opportunely and prevent further organ damage.

Regarding the manifestations of the acute phase, nonspecific symptoms such as diarrhea, emesis, and persistent cough, or one of the pathognomonic signs in cases of acute CD—the chagoma of inoculation—were unrecognized by the majority of participants. These have been observed in acute cases in the Brazilian Amazon [32]. On case is related to a 2-year-old child who died from acute chagasic myocarditis after presenting with fever, abdominal pain, headache, and vomiting [33], or in a diagnosis of the disease 40 years after the manifestation of an unusual febrile illness, marked facial swelling, and chronic dry cough [34].

Despite the low incidence of CD in endemic areas, the increase in outbreaks due to oral transmission requires attention from health professionals [28] in the investigation of apparently nonspecific signs/symptoms, not only for the early treatment of the affected person to prevent disease progression but also to trigger an epidemiological investigation with the timely identification of the source of infection, the magnitude of the outbreak, and the delineation of risk areas.

Regarding the treatment of CD, the majority of workers considered it incurable, with no percentage differences among occupational categories, which demonstrates the real absence of univocal clinical criteria to classify a person as cured. On the other hand, for acute cases, the so-called serological cure—seronegativation—is used, which requires the immediate implementation of antiparasitic treatment, recommended by the WHO for the acute and recent chronic phases [1].

Although the workers acknowledged the existence of etiological treatment, the majority did not know the indicated drug, with the exception of physicians and nurses who reported knowing it. Studies conducted in Brazil, Argentina, and Colombia revealed physicians’ lack of knowledge regarding the etiological treatment of CD [13,35,36], with no such information found for the other occupational categories in the consulted literature. Therefore, it is possible that the lack of knowledge about the treatment is responsible for only 1% of cases being treated annually [2].

The recognized antiparasitic medication was benznidazole among all categories of workers. It is the first-choice drug, indicated in secondary prevention to avoid organ damage or its progression, as well as to prevent the carrier from becoming a source of infection [37]. Its use in the acute phase of the disease cures 80% of diagnosed individuals and can reach 100.0% in cases of congenital transmission [38].

Nifurtimox, in turn, is the second-line drug for all acute or chronic cases in the indeterminate or digestive forms in children and adolescents when there is therapeutic failure or adverse effects from benznidazole, although it was not recognized as such by the participants of the present study. Etiological treatment with benznidazole is indicated in all acute and chronic cases in individuals under 50 years of age with indeterminate and digestive manifestations, and in all age groups in the initial stages of the cardiac form [27].

Sustained vector-borne transmission of *T*. *cruzi* remains active in all endemic areas. The subfamily Triatominae (Hemiptera and Reduviidae) is composed of 158 species and 18 genera [39]. In Brazil, the northeast region stands out for its rich diversity and wide dispersion of triatomines, and the state of Bahia has 26 of these species [40,41]. In the municipality of Irecê, triatomine bugs fed on human blood infected with *T. cruzi* have been identified, thereby reinforcing the potential for sustained parasite transmission [42].

Thus, the recognition of triatomine bugs is fundamental for the effectiveness of entomological surveillance, and the present results revealed that the majority of health professionals claimed to be able to identify them, with *Triatoma infestans* being the most frequently recognized species. However, the majority did not recognize the 4^th^ nymph stage of *Rhodnius* sp and this finding was consistent across all professional categories. This contrasts with reports from two CD-free locations where physicians’ knowledge was greater compared to other professionals [13]. Therefore, it is important to emphasize that the immature forms of triatomine bugs can transmit *T*. *cruzi*, and their presence within the home indicates probable colonization of this environment [43].

Since the elimination of vector-borne transmission seems unfeasible in South America due to the multiplicity of wild mammals that are natural reservoirs of *T*. *cruzi* and the existence of more than 100 vector species [44], the recognition of triatomine bugs by health professionals is essential. This knowledge underprints effective community health education to strengthen passive entomological surveillance actions through population participation identifying the presence of triatomine bugs in their residence and communicating this to health services, in addition to being guided on care in handling and where to forward the suspected specimens.

Regarding the handling of the insects, the participants of the present study recognized that hands need to be protected with gloves or plastic bags as advocated in technical manuals and by the Ministry of Health [45]. On the other hand, the majority of workers indicated that the place to forward the insects is the Zoonoses Control Center, instead of the PITs—reference sites for the delivery of triatomine bugs [12], despite there being 11 in the studied municipality.

For effective surveillance of triatomine bugs, it is fundamental to implement PITs in urban areas of the municipality, located in places easily accessible to the population, such as health services or surveillance sectors. In rural areas, PITs should be installed in strategically important localities from an epidemiological point of view, and can operate in schools, health units, residences, or commercial establishments, under the responsibility of volunteers, community leaders, or residents [46]. Considering the potential demands generated by the PITs, it is essential that community health agents receive continuous training, due to their direct contact with the population and the critical role they play in household-level interventions.

The majority of the interviewed professionals, regardless of occupational category, identified that people bitten by a triatomine bug need to be monitored by serology, which is aligns with the recommendations of the Brazilian Ministry of Health [45]. This is in addition to submission to parasitological exams as they are suspected cases of acute CD. Acute cases, even if suspected, can be considered sentinel events that mandatorily trigger active surveillance actions to identify the magnitude and geographical distribution of the disease, probable sources of infection, and the presence of triatomine bugs in the peridomicile and intradomiciliary areas.

For accurate surveillance in the CD situation, which also includes diagnosis and treatment, the need for access to information by health workers is reiterated. In the context of the present study, this occurred informally in the daily work routine, particularly for community health agents, or during health-related training courses for physicians, nurses, and nursing assistants/technicians.

In the case of professionals with specific training in the health field, particularly at the higher education level, CD should be a mandatory subject in Brazil due to its endemicity. This is necessary to ensure professionals can improve diagnosis, since about 70% of those infected by *T*. *cruzi* are unaware of their condition [2], as well as to provide etiological treatment when indicated, follow-up of chronic cases, and appropriate management of clinical manifestations.

On the other hand, the need to qualify the multiprofessional teams working in primary care is assumed, consistent with the occupation exercised, with particular attention to community health agents, who are daily in the homes of the registered population and, for this reason, need a keen eye for identifying potential cases of CD, as well as in recognizing triatomine bugs.

For the prevention and control of CD, as it is a disease whose epidemiological chain has many and diverse elements, such as multiple vector species, animal reservoirs, and a strong influence of environmental and social factors, its approach needs to be broad, considering human, animal, and environmental health, converging with the One Health perspective [47].

Factors such as deforestation, unplanned urbanization, and changes in land use increase the risk of CD transmission, requiring intersectoral actions that involve integrated surveillance, vector control, ecological monitoring, and improvements in the living conditions of affected populations [48].

Despite the precautions taken to avoid selection bias of the loss type—three visits to the unit in search of the health professional—it cannot be disregarded that it may have occurred. Furthermore, even with the training of interviewers, observer bias may have occurred when the researcher emphasizes questions considered of interest for the study more than others, or social desirability bias, when the participant responds according to what is socially accepted. As the participant selection method did not involve randomization, inferential statistical techniques were not used, which prevented the identification of factors statistically associated with the professionals’ knowledge.

It is important to emphasize that several works in the scientific literature have consistently pointed to the urgent need to develop continuing education programs aimed at improving the knowledge of health professionals about other neglected diseases. A study conducted in the city of Juiz de Fora, in the state of Minas Gerais (Brazil), through the application of a structured questionnaire on “Q Fever,” revealed worrying data: 92.9% of the evaluated physicians were unaware of the said disease, and only 3 professionals (1.2%) answered more than half of the specific questions about the disease correctly [49].

In another study with health professionals, conducted in the city of Peshawar (Pakistan), significant gaps in knowledge about “Rabies,” its forms of transmission, prevention, and treatment were revealed. Although the majority recognized that the disease is transmitted by dog bites, only a portion of the interviewees knew about the possibility of transmission through scratches, licks, or even contaminated water. There were also significant doubts about the use and availability of anti-rabies vaccines (ARV) and rabies immunoglobulin (RIG), especially among paramedics and early-career physicians [50]. A study conducted in northwestern Ethiopia with 384 health professionals revealed that only 28.13% had good knowledge about Mpox, none of the participants had received formal training on Mpox, and 100% reported never having been vaccinated against Mpox or smallpox in the last five years. These data highlight serious gaps in the preparedness of professionals, which compromises the response to outbreaks and public health emergencies [51].

## Conclusion

The present study is one of the few conducted in Brazil and worldwide whose object was the knowledge about CD among health professionals in multidisciplinary teams in PHC. It can be said that the knowledge of PHC workers about CD in the studied municipality—which may reflect the reality of Brazil—is incipient and differs among occupational categories, which can impact the quality of surveillance actions, which range from raising a diagnostic hypothesis to the implementation of prevention and control measures.

To make CD surveillance effective in PHC, it is necessary to promote the inclusion of CD in the curricula of vocational and undergraduate health courses, as well as to encourage continuing education activities (i.e., lectures, training, theoretical-practical courses), with particular emphasis on nonspecific acute phase signs/symptoms such as persistent fever, diarrhea, emesis, or pathognomonic ones (i.e., chagoma of inoculation); modes of transmission less common than vector-borne; etiological treatment and its indication; management of suspected cases; identification of triatomine species and the locations for forwarding suspected specimens; as well as other aspects such as the criteria for notification of acute and chronic CD cases and epidemiological investigation.

Studies with PHC professionals in municipalities from different regions of the country are recommended, including endemic and non-endemic locations for CD. Furthermore, probabilistic samples are recommended so that inferential statistical techniques can be employed.

## Supporting information

S1 Appendix Consent Form (ICF). (DOCX)

S2 Appendix Health workers’ knowledge questionnaire about Chagas disease. (PDF)

S3 Appendix Figures of kissing bugs presented to identify the specimes. (TIF)

S4 Appendix Complete dataset supporting the presented analyses. (XLSX)
