# Supplementary material for: Knowledge about Chagas disease among Primary Health Care professionals in a municipality located in northeastern Brazil
Source: PLoS Negl Trop Dis. 2026 Feb 9;20(2):e0014000. doi: 10.1371/journal.pntd.0014000 (PMC12981557; doi:10.1371/journal.pntd.0014000)
Supplement: S1 Appendix — (DOCX) [file pntd.0014000.s001.docx]

CONSENT FORM (ICF)

You are invited to participate anonymously in the study entitled "Epidemiological survey for Chagas disease in areas vulnerable to vector transmission in the Identity Territory of Irecê, Bahia". The general objective of this study is to investigate the possible existence of sustained vector transmission of *Trypanosoma cruzi* (*T. cruzi*) in priority areas of the Irecê Identity Territory, Bahia. The sub-project entitled " **Knowledge about Chagas disease among primary health care professionals in a municipality located in northeastern Brazil**" will assess the knowledge among health professionals regarding the biology of the parasite-vector-host of Chagas disease. In order to take part in this research, you will be asked to sign two original copies of this consent form (one copy for you and one for the researcher), and to complete a questionnaire containing questions about Chagas' disease as well as items on personal information and socio-economic information.

The possible **risks** include: invasion of privacy, disclosure of confidential information (recorded in this ICF), discrimination and stigmatization based on the content revealed and minor complications such as vein or vessel inflammation. To reduce invasion of privacy, you will answer the questionnaire at your chosen place of work or residence. To ensure that your data and answers are not disclosed, only the researchers will have access to your identity, and you will be recognized in the database by a computer-generated code. The **benefits** for those taking part in this research will be **indirect.** The knowledge generated will contribute to advancing scientific understanding and supporting future research on Chagas disease, with potential implications for public health strategies. The principal investigator will give access to the results to all study participants.

There will be no monetary remuneration for participation. However, participants will be duly **reimbursed** for any expenses incurred as a result of taking part iin this research, such as food, transportation and materials. This will be done upon request tothe principal investigator with appropriate documentation, using funds allocated to the project.It is important to note that this research aims to publish the results in scientific journals, and the data collected will be securely stored and managed through RedCap platform^TM^  (Research Electronic Data Capture) . The platform is hosted at the Gonçalo Moniz Institute - Fiocruz/BA. You are free to decline participation or to to withdraw your consent at any stage of the study, without any negative consequences.
