# Supplementary material for: Knowledge about Chagas disease among Primary Health Care professionals in a municipality located in northeastern Brazil
Source: PLoS Negl Trop Dis. 2026 Feb 9;20(2):e0014000. doi: 10.1371/journal.pntd.0014000 (PMC12981557; doi:10.1371/journal.pntd.0014000)
Supplement: S2 Appendix — (PDF) [file pntd.0014000.s002.pdf]

Data Dictionary Codebook

	#	Variable / Field Name	Field Label<br>*Field Note*	Field Attributes (Field Type, Validation, Choices, Calculations, etc.)
	1	[record_id]	Record ID	text
	2	[date]	Data da entrevista	text (datetime_dmy), Required
	3	[ninv]	Entrevistador (a)	radio, Required<br>1 Márcio Almeida<br>2 Jorgana Soares

Instrument: Questionário conhecimento dos trabalhadores da saúde sobre Doença de Chagas (questionrio_conhecimento_dos_trabalhadores_da_sade)

	#	Variable / Field Name	Field Label	Field Attributes
	4	[codubsf]	Unidade de lotação	radio, Required<br>1 Amador Aguiar<br>2 Antônio Carlos Ribeiro<br>3 Antônio Carlos Souza Diniz<br>4 Ana Mendes<br>5 Arnobio Batista<br>6 Benedito Ney<br>7 Camila Gomes (Vivendas II)<br>8 Édiçon Ribeiro dos Santos<br>9 Ênio Rosendo Pinto<br>10 ina Gomes de Oliveira<br>11 Helma Cristiane C. de Oliveira<br>12 Horácio Fernandes da Fonseca<br>13 Iêda I, II e III<br>14 Indalécio Vanderlei Soares<br>15 José Antônio de Souza<br>16 Lagoa Nova<br>17 Newton Marques Dourado<br>18 Raimundo C. Sombra<br>19 Sinézia Caldeira Bella<br>20 Sinval Cesar Vasconcelos<br>21 Valdomiro Galdino da Silva<br>22 Vasconcelos (Vivendas I)<br>99 Ignorado
	5	[nome]	Section Header: *Bloco 1- Características sociodemográficas Nesse bloco, o (a) Sr (a) será perguntado sobre dados que lhe identificam como pessoa:*<br>Qual é o seu nome?	text, Required
	6	[datn]	Qual é a sua data de nascimento?	text (date_dmy), Required
	7	[idad]	Idade	calc, Required<br>Calculation: if(rounddown (datediff([datn], [date], "y", "dmy", true),0)=0,1,rounddown (datediff([datn], [date], "y", "dmy", true),0))
	8	[gen]	Qual é o seu gênero?	radio, Required<br>1 Feminino<br>2 Masculino<br>3 Não binário<br>9 Ignorado

	9	[corp]	Qual é a cor da sua pele?	radio, Required<br>0 Branca<br>1 Parda<br>2 Negra

				3 Amarela/Indígena/Oriental<br>9 Ignorado
	10	[lnas]	Onde o (a) Sr (a) nasceu?	radio, Required<br>1 Irecê<br>2 Outra cidade<br>9 Ignorado<br><br>Field Annotation: Qual local?
	11	[qlna]<br><br>Show the field ONLY if:<br>[lnas] = '2'	Qual cidade?	text, Required
	12	[lmor]	Onde o (a) Sr (a) mora?	radio, Required<br>1 Irecê<br>2 Outra cidade<br>9 Ignorado
	13	[qlmo]<br><br>Show the field ONLY if:<br>[lmor] = '2'	Qual cidade?	text
	14	[mbtr]	O (a) Sr (a) mora no mesmo bairro em que trabalha?	radio, Required<br>0 Sim<br>1 Não<br>9 Ignorado
	15	[esco]	Até que série o (a) Sr (a) estudou?	radio, Required<br>0 Não alfabetizado (a)<br>1 1° grau/Ensino Fundamental incompleto<br>2 1° grau/Ensino Fundamental completo<br>3 2° grau/Ensino Médio incompleto<br>4 2° grau/Ensino Médio completo<br>5 3° grau/Ensino Superior incompleto<br>6 3° grau/Ensino Superior Completo<br>7 Especialização<br>8 Mestrado/Doutorado<br>9 Ignorado
	16	[rfam]	Qual é a renda mensal da sua família (R$)?	text (number_2dp), Required
	17	[ocup]	Section Header: *Bloco 2 - Características do Trabalho Nesse bloco, o (a) Sr (a) será perguntado sobre dados que estão relacionados com o seu trabalho remunerado:*<br><br>Qual é a sua ocupação?	radio, Required<br>0 Médico (a)<br>1 Enfermeiro (a)<br>2 Técnico (a)/Auxiliar de Enfermagem<br>3 Agente Comunitário (a) de Saúde/Agente de Controle de Endemias<br>4 Cirurgião (ã) dentista/odontólogo (a)<br>5 Auxiliar de consultório dentário/técnico (a) de higiene dental<br>6 Auxiliar administrativo/recepcionista<br>7 Vigilante<br>8 Outro (a)<br>9 Ignorado
	18	[ouoc]<br><br>Show the field ONLY if:<br>[ocup] = '6'	Ocupação	text
	19	[ttra]	Há quantos anos o (a) Sr (a) trabalha na sua ocupação?	text (number), Required

	20	[jort]	Qual é a sua jornada de trabalho semanal nessa Unidade de Saúde da Família?	radio, Required<br>1 Até 20 horas<br>2 Entre 20 a 40 horas<br>3 40 horas ou mais<br>9 Ignorado
	21	[tusf]	Há quantos anos o (a) Sr (a) trabalha nessa Unidade de Saúde da Família?	text (number), Required
	22	[tvin]	Qual é o seu vínculo empregatício com a prefeitura?	radio, Required<br>1 Empregado (a) com carteira assinada<br>2 Empregado (a) sem carteira assinada/REDA<br>3 Pessoa jurídica/autônomo/conta própria<br>4 Servidor (a) público (a) estatuário (a)<br>5 Servidor (a) público (a) celetista<br>6 Cooperativado (a)<br>9 Ignorado
	23	[trsf]	O (a) Sr. (a) trabalhou anteriormente na Estratégia de Saúde da Família?	radio, Required<br>0 Sim<br>1 Não<br>9 Ignorado
	24	[cbar]	Section Header: *Bloco 3 - Conhecimento sobre a Doença de Chagas*<br>*Nesse bloco, o (a) Sr (a) será perguntado sobre o seu conhecimento acerca da Doença de Chagas.*<br>O (a) Sr (a) conhece o inseto "barbeiro"?	radio, Required<br>0 Sim<br>1 Não<br>9 Ignorado
	25	[idba]<br>Show the field ONLY if:<br>[cbar] = '0'	Identifique, qual (is) do (s) inseto (s) é (são) "barbeiro (s)/chupança (s)":	checkbox, Required<br>1 idba___1 Inseto a<br>2 idba___2 Inseto b<br>3 idba___3 Inseto c<br>4 idba___4 Inseto d<br>5 idba___5 Inseto e<br>6 idba___6 Nenhum deles<br>9 idba___9 Ignorado
	26	[acdch]	Quem causa a Doença de Chagas?	radio, Required<br>0 Vírus<br>1 Bactéria<br>2 Fungo<br>3 Protozoário<br>4 Não sei informar<br>9 Ignorado
	27	[trand]	Qual (is) (são) a(s) forma(s) de transmissão da Doença de Chagas?	checkbox, Required<br>0 trand___0 Transfusão sanguínea<br>1 trand___1 Acidentes com sangue/material biológico<br>2 trand___2 Transmissão de mãe para filho<br>3 trand___3 Comer alimentos contaminados<br>4 trand___4 Picada do barbeiro/chupança<br>5 trand___5 Não sei dizer<br>9 trand___9 Ignorado

	28	[podch]	Identifique qual (is) é (são) o (s) o(s) principal (is) órgão (s) que a Doença de Chagas acomete:	checkbox, Required<br>1 | podch___1 | Coração<br>2 | podch___2 | Baço<br>3 | podch___3 | Intestino Grosso<br>4 | podch___4 | Cérebro<br>5 | podch___5 | Esôfago<br>6 | podch___6 | RIM<br>7 | podch___7 | Fígado<br>8 | podch___8 | Não sei informar<br>9 | podch___9 | Ignorado
	29	[sagu]	Identifique qual (is) é (são) o (s) sintoma (s) que a pessoa com a Doença de Chagas apresenta na fase aguda:	checkbox<br>1 | sagu___1 | Febre<br>2 | sagu___2 | Aparecimento de ínguas<br>3 | sagu___3 | Diarreia<br>4 | sagu___4 | Inchaço/ edema<br>5 | sagu___5 | Vômito<br>6 | sagu___6 | Aumento do baço<br>7 | sagu___7 | Aumento do fígado<br>8 | sagu___8 | Tosse persistente<br>9 | sagu___9 | Olho inchado (Sinal de Romaña)<br>10 | sagu___10 | Furúnculo (Chagoma de Inoculação)<br>11 | sagu___11 | Não sei informar<br>99 | sagu___99 | Ignorado
	30	[scro]	Identifique qual (is) é (são) o (s) sinal (is) sintoma (s) que a pessoa apresenta na fase crônica:	checkbox, Required<br>1 | scro___1 | Alterações Eletrocardiográficas<br>2 | scro___2 | Megacólon<br>3 | scro___3 | Megaesôfago<br>4 | scro___4 | Insuficiência Cardíaca Congestiva<br>5 | scro___5 | Fenômenos Tromboembólicos<br>6 | scro___6 | Pode não apresentar sintomas<br>7 | scro___7 | Desconforto respiratório<br>8 | scro___8 | Não sei informar<br>9 | scro___9 | Ignorado
	31	[trae]	A doença de Chagas tem tratamento específico para a sua causa?	radio, Required<br>1 | Sim<br>2 | Não<br>3 | Não sei informar<br>4 | Ignorado
	32	[medc]<br>Show the field ONLY if:<br>[trae] = '1'	Qual (is) medicamento (s) é (são) recomendado (s) para tratar a causa da Doença de Chagas?	checkbox, Required<br>1 | medc___1 | Benzonidazol<br>2 | medc___2 | Nifirtimox<br>3 | medc___3 | Amiodarona<br>4 | medc___4 | Propranolol<br>5 | medc___5 | Não sei informar<br>9 | medc___9 | Ignorado
	33	[cura]	A doença de Chagas tem cura?	radio, Required<br>0 | Sim<br>1 | Não<br>2 | Não sei informar<br>9 | Ignorado

	34	[orien]	Como o (a) Sr. (a) orientaria uma pessoa que encontrou um inseto "barbeiro/chupança" na sua residência?	radio, Required<br>1 Matar o inseto e eliminar o resíduo com limpeza profunda<br>2 Soltar o inseto em uma mata próxima<br>3 Nada se deve fazer<br>4 Não sei informar<br>9 Ignorado
	35	[manip]	Caso seja necessário manipular o "barbeiro/chupança", como esse processo deve ser feito?	radio, Required<br>1 Não precisa proteger as mãos para isso<br>2 Luvas ou proteção com saco plástico<br>3 Não devemos manipular barbeiro em nenhuma hipótese<br>4 Não sei informar<br>9 Ignorado
	36	[encam]	Para qual (is) serviço (s) o (a) Sr (a) pode encaminhar o inseto barbeiro/chupança?	checkbox, Required<br>1 encam___1 Posto de Informação de Triatomíneos (PIT)<br>2 encam___2 Centro de Controle de Zoonoses (CCZ)<br>3 encam___3 Secretaria Municipal de saúde (SMS)<br>4 encam___4 Não sei informar<br>5 encam___5 Ignorado
	37	[pproc]	Caso uma pessoa seja picada pelo inseto barbeiro/chupança, o que o (a) Sr (a) orientaria?	radio, Required<br>1 Fazer testes sanguíneos e acompanhar a sorologia<br>2 Iniciar o tratamento para a doença imediatamente<br>3 Aguardar o aparecimento de sintomas<br>4 Não sei informar<br>9 Ignorado
	38	[info]	Como o (a) Sr (a) teve acesso à informação sobre doença de Chagas?	radio, Required<br>1 Durante a graduação/ensino profissionalizante<br>2 Especialização<br>3 Mestrado<br>4 Doutorado<br>5 Curso de capacitação<br>6 Outro (a)<br>7 Nunca obtive informação alguma sobre Doença de Chagas<br>9 Ignorado
	39	[qidc]<br>Show the field ONLY if:<br>[info] = '6'	Qual a outra maneira pela qual obteve informação sobre Doença de Chagas?	radio, Required
	40	[rcam]	Section Header: *Bloco 4 - Práticas concernentes à Doença de Chagas desenvolvidas pelo setor saúde Nesse bloco, o (a) Sr (a) será perguntado sobre as Práticas desenvolvidas no setor saúde do município em que trabalha referentes à Doença da Chagas.*<br>A Secretaria Municipal de Saúde de Irecê realiza campanhas para a prevenção da Doença de Chagas?	radio, Required<br>0 Sim<br>1 Não<br>2 Não sei informar<br>9 Ignorado
	41	[ecam]	A Secretaria Municipal de Saúde de Irecê estimula a realização de campanhas para a prevenção da Doença de Chagas na Unidade de Saúde da Família em que o (a) Sr. (a) trabalha?	radio, Required<br>0 Sim<br>1 Não<br>2 Não sei informar<br>9 Ignorado

	42	[prev]	Existe algum programa de prevenção à Doença de Chagas na Unidade de Saúde da Família em que o (a) Sr. (a) trabalha?	radio, Required<br>0 Sim<br>1 Não<br>2 Não sei informar<br>9 Ignorado
	43	[ppeu]<br>Show the field ONLY if:<br>[prev] = '0'	Qual é o programa de prevenção para Doença de Chagas existente na USF?	text, Required
	44	[matdi]	Na Unidade de Saúde da Família em que o (a) Sr (a) trabalha está disponível algum tipo de material para a orientação em casos suspeitos de Doença de Chagas, incluindo como proceder quando encontrar insetos perto ou dentro de casa?	radio, Required<br>1 Sim<br>2 Não<br>3 Não sei<br>9 Ignorado
	45	[bbic]	A equipe em que o (a) Sr (a) trabalha realiza busca por barbeiros no interior das casas no território de abrangência da Unidade de Saúde da Família?	radio, Required<br>0 Sim<br>1 Não<br>2 Não sei<br>9 Ignorado
	46	[bbrc]	A equipe em que o (a) Sr (a) trabalha realiza busca por barbeiros ao redor das casas no território de abrangência da Unidade de Saúde da Família?	radio, Required<br>0 Sim<br>1 Não<br>2 Não sei<br>9 Ignorado
	47	[pren]	Na Unidade de Saúde da Família em que o (a) Sr (a) trabalha é realizada a pesquisa para a Doença de Chagas durante o atendimento pré-natal ?	radio, Required<br>0 Não<br>1 Sim<br>2 Não sei informar<br>9 Ignorado
	48	[rbtr]	O (a) Sr (a) já recebeu de alguma pessoa um inseto "barbeiro/chupança" na Unidade de Saúde da Família onde trabalha?	radio, Required<br>0 Não<br>1 Sim<br>2 Não sei<br>9 Ignorado
	49	[slba]	Na Unidade de Saúde da Família em que o (a) Sr (a) trabalha é realizada a coleta de sangue para a sorologia de Doença de Chagas quando é localizado pelo usuário ou pela equipe um "barbeiro/chupança"?	radio, Required<br>0 Sim<br>1 Não<br>2 Não sei<br>9 Ignorado
	50	[atpb]	O (a) Sr (a) já atendeu na Unidade de Saúde da Família em que trabalha pessoas picadas pelo "barbeiro/chupança"?	radio, Required<br>0 Não<br>1 Não lembro<br>2 Não sei informar<br>3 Sim<br>9 Ignorado
	51	[sdch]	O (a) Sr (a) já suspeitou de Doença de Chagas em alguma pessoa atendida na Unidade de Saúde da Família em que trabalha?	radio, Required<br>0 Não<br>1 Sim<br>2 Não sei informar<br>9 Ignorado

	52	[diag]	Alguma pessoa foi diagnosticada com doença de Chagas na Unidade de Saúde da Família em que o (a) Sr. (a) trabalha?	radio, Required<br>0 Não<br>1 Sim<br>2 Não sei informar<br>9 Ignorado
	53	[atdc]	Na Unidade de Saúde da Família em que o (a) Sr. (a) trabalha é realizado o atendimento às pessoas portadoras de Doença de Chagas?	radio, Required<br>0 Sim<br>1 Não<br>2 Não sei informar<br>9 Ignorado
	54	[mopc]<br><br>Show the field ONLY if:<br>[atdc] = '0'	Qual é o tipo de moradia das pessoas atendidas com Doença de Chagas na Unidade de Saúde da Família em que o (a) Sr (a) trabalha?	radio, Required<br>0 Tijolo/adobe<br>1 Madeira<br>2 Taipa revestida<br>3 Taipa não revestida<br>4 Material aproveitado<br>5 Não sei informar<br>9 Ignorado
	55	[baid]<br><br>Show the field ONLY if:<br>[atdc] = '0'	A equipe da Unidade de Saúde da Família em que o (a) Sr (a). trabalha procura por "barbeiros" dentro da casa das pessoas portadoras de Doença de Chagas aqui atendidas?	radio, Required<br>0 Sim<br>1 Não<br>2 Não sei informar<br>9 Ignorado
	56	[bapd]<br><br>Show the field ONLY if:<br>[atdc] = '0'	A equipe da Unidade de Saúde da Família em que o (a) Sr (a). trabalha procura por "barbeiros" ao redor da casa das pessoas portadoras de Doença de Chagas aqui atendidas?	radio, Required<br>0 Sim<br>1 Não<br>2 Não sei informar<br>9 Ignorado
	57	[questionrio_conhecimento_dos_trabalhadores_da_sade_complete]	Section Header: *Form Status*<br>Complete?	dropdown<br>0 Incomplete<br>1 Unverified<br>2 Complete
Instrument: **Questionário conhecimento dos trabalhadores da saúde sobre Doença de Chagas 2 (questionrio_conhecimento_dos_trabalhadores_da_c7f8)**				
	58	[date_v2]	Data da entrevista	text (datetime_dmy), Required
	59	[ninv_v2]	Entrevistador (a)	radio, Required<br>1 Márcio Almeida<br>2 Jorgana Soares

	60	[codubsf_v2]	Unidade de lotação	radio, Required<br>1 Amador Aguiar<br>2 Antônio Carlos Ribeiro<br>3 Antônio Carlos Souza Diniz<br>4 Ana Mendes<br>5 Arnobio Batista<br>6 Benedito Ney<br>7 Camila Gomes (Vivendas II)<br>8 Édiçon Ribeiro dos Santos<br>9 Ênio Rosendo Pinto<br>10 ina Gomes de Oliveira<br>11 Helma Cristiane C. de Oliveira<br>12 Horácio Fernandes da Fonseca<br>13 Iêda I, II e III<br>14 Indalécio Vanderlei Soares<br>15 José Antônio de Souza<br>16 Lagoa Nova<br>17 Newton Marques Dourado<br>18 Raimundo C. Sombra<br>19 Sinézia Caldeira Bella<br>20 Sinval Cesar Vasconcelos<br>21 Valdomiro Galdino da Silva<br>22 Vasconcelos (Vivendas I)<br>99 Ignorado
	61	[nome_v2]	Section Header: *Bloco 1- Características sociodemográficas Nesse bloco, o (a) Sr (a) será perguntado sobre dados que lhe identificam como pessoa:*<br>Qual é o seu nome?	text, Required
	62	[datn_v2]	Qual é a sua data de nascimento?	text (date_dmy), Required
	63	[idad_v2]	Idade	calc, Required<br>Calculation: if(rounddown (datediff([datn_v2], [date_v2], "y", "dmy", true),0)=0,1,rounddown (datediff([datn_v2], [date_v2], "y", "dmy", true),0))
	64	[gen_v2]	Qual é o seu gênero?	radio, Required<br>1 Feminino<br>2 Masculino<br>3 Não binário<br>9 Ignorado
	65	[corp_v2]	Qual é a cor da sua pele?	radio, Required<br>0 Branca<br>1 Parda<br>2 Negra<br>3 Amarela/Indígena/Oriental<br>9 Ignorado
	66	[lnas_v2]	Onde o (a) Sr (a) nasceu?	radio, Required<br>1 Irecê<br>2 Outra cidade<br>9 Ignorado<br>Field Annotation: Qual local?
	67	[qlna_v2]<br>Show the field ONLY if:<br>[lnas_v2] = '2'	Qual cidade?	text, Required

	68	[lmor_v2]	Onde o (a) Sr (a) mora?	radio, Required<br>1 Irecê<br>2 Outra cidade<br>9 Ignorado
	69	[qlmo_v2]<br>Show the field ONLY if:<br>[lmor_v2] = '2'	Qual cidade?	text
	70	[mbtr_v2]	O (a) Sr (a) mora no mesmo bairro em que trabalha?	radio, Required<br>0 Sim<br>1 Não<br>9 Ignorado
	71	[esco_v2]	Até que série o (a) Sr (a) estudou?	radio, Required<br>0 Não alfabetizado (a)<br>1 1º grau/Ensino Fundamental incompleto<br>2 1º grau/Ensino Fundamental completo<br>3 2º grau/Ensino Médio incompleto<br>4 2º grau/Ensino Médio completo<br>5 3º grau/Ensino Superior incompleto<br>6 3º grau/Ensino Superior Completo<br>7 Especialização<br>8 Mestrado/Doutorado<br>9 Ignorado
	72	[rfam_v2]	Qual é a renda mensal da sua família (R$)?	text (number_2dp), Required
	73	[ocup_v2]	Section Header: *Bloco 2 - Características do Trabalho Nesse bloco, o (a) Sr (a) será perguntado sobre dados que estão relacionados com o seu trabalho remunerado:*<br>Qual é a sua ocupação?	radio, Required<br>0 Médico (a)<br>1 Enfermeiro (a)<br>2 Técnico (a)/Auxiliar de Enfermagem<br>3 Agente Comunitário (a) de Saúde/Agente de Controle de Endemias<br>4 Cirurgião (ã) dentista/odontólogo (a)<br>5 Auxiliar de consultório dentário/técnico (a) de higiene dental<br>6 Auxiliar administrativo/recepcionista<br>7 Vigilante<br>8 Outro (a)<br>9 Ignorado
	74	[ouoc_v2]<br>Show the field ONLY if:<br>[ocup_v2] = '6'	Ocupação	text
	75	[ttra_v2]	Há quantos anos o (a) Sr (a) trabalha na sua ocupação?	text (number), Required
	76	[jort_v2]	Qual é a sua jornada de trabalho semanal nessa Unidade de Saúde da Família?	radio, Required<br>1 Até 20 horas<br>2 Entre 20 a 40 horas<br>3 40 horas ou mais<br>9 Ignorado
	77	[tusf_v2]	Há quantos anos o (a) Sr (a) trabalha nessa Unidade de Saúde da Família?	text (number), Required

	78	[tvin_v2]	Qual é o seu vínculo empregatício com a prefeitura?	radio, Required<br>1 Empregado (a) com carteira assinada<br>2 Empregado (a) sem carteira assinada/REDA<br>3 Pessoa jurídica/autônomo/conta própria<br>4 Servidor (a) público (a) estatuário (a)<br>5 Servidor (a) público (a) celetista<br>6 Cooperativado (a)<br>7 Outro<br>9 Ignorado
	79	[esovi]<br>Show the field ONLY if:<br>[tvin_v2] = '7'	Especificação de outro vínculo	text
	80	[trsf_v2]	O (a) Sr. (a) trabalhou anteriormente na Estratégia de Saúde da Família?	radio, Required<br>0 Sim<br>1 Não<br>9 Ignorado
	81	[cbar_v2]	Section Header: *Bloco 3 - Conhecimento sobre a Doença de Chagas*<br>*Nesse bloco, o (a) Sr (a) será perguntado sobre o seu conhecimento acerca da Doença de Chagas.*<br>O (a) Sr (a) conhece o inseto "barbeiro"?	radio, Required<br>0 Sim<br>1 Não<br>9 Ignorado
	82	[idba_v2]<br>Show the field ONLY if:<br>[cbar_v2] = '0'	Identifique, qual (is) do (s) inseto (s) é (são) "barbeiro (s)/chupança (s)":	checkbox, Required<br>1 idba_v2___1 Inseto a<br>2 idba_v2___2 Inseto b<br>3 idba_v2___3 Inseto c<br>4 idba_v2___4 Inseto d<br>5 idba_v2___5 Inseto e<br>6 idba_v2___6 Nenhum deles<br>9 idba_v2___9 Ignorado
	83	[acdch_v2]	Quem causa a Doença de Chagas?	radio, Required<br>0 Vírus<br>1 Bactéria<br>2 Fungo<br>3 Protozoário<br>4 Não sei informar<br>9 Ignorado
	84	[trand_v2]	Qual (is) (são) a(s) forma(s) de transmissão da Doença de Chagas?	checkbox, Required<br>0 trand_v2___0 Transfusão sanguínea<br>1 trand_v2___1 Acidentes com sangue/material biológico<br>2 trand_v2___2 Transmissão de mãe para filho<br>3 trand_v2___3 Comer alimentos contaminados<br>4 trand_v2___4 Picada do barbeiro/chupança<br>5 trand_v2___5 Não sei dizer<br>9 trand_v2___9 Ignorado

	85	[podch_v2]	Identifique qual (is) é (são) o (s) o(s) principal (is) órgão (s) que a Doença de Chagas acomete:	checkbox, Required<br>1 podch_v2___1 Coração<br>2 podch_v2___2 Baço<br>3 podch_v2___3 Intestino Grosso<br>4 podch_v2___4 Cérebro<br>5 podch_v2___5 Esôfago<br>6 podch_v2___6 RIM<br>7 podch_v2___7 Fígado<br>8 podch_v2___8 Não sei informar<br>9 podch_v2___9 Ignorado
	86	[sagu_v2]	Identifique qual (is) é (são) o (s) sintoma (s) que a pessoa com a Doença de Chagas apresenta na fase aguda:	checkbox<br>1 sagu_v2___1 Febre<br>2 sagu_v2___2 Aparecimento de ínguas<br>3 sagu_v2___3 Diarreia<br>4 sagu_v2___4 Inchaço/ edema<br>5 sagu_v2___5 Vômito<br>6 sagu_v2___6 Aumento do baço<br>7 sagu_v2___7 Aumento do fígado<br>8 sagu_v2___8 Tosse persistente<br>9 sagu_v2___9 Olho inchado (Sinal de Romaña)<br>10 sagu_v2___10 Furúnculo (Chagoma de Inoculação)<br>11 sagu_v2___11 Não sei informar<br>99 sagu_v2___99 Ignorado
	87	[scro_v2]	Identifique qual (is) é (são) o (s) sinal (is) sintoma (s) que a pessoa apresenta na fase crônica:	checkbox, Required<br>1 scro_v2___1 Alterações Eletrocardiográficas<br>2 scro_v2___2 Megacólon<br>3 scro_v2___3 Megaesôfago<br>4 scro_v2___4 Insuficiência Cardíaca Congestiva<br>5 scro_v2___5 Fenômenos Tromboembólicos<br>6 scro_v2___6 Pode não apresentar sintomas<br>7 scro_v2___7 Desconforto respiratório<br>8 scro_v2___8 Não sei informar<br>9 scro_v2___9 Ignorado
	88	[trae_v2]	A doença de Chagas tem tratamento específico para a sua causa?	radio, Required<br>1 Sim<br>2 Não<br>3 Não sei informar<br>4 Ignorado
	89	[medc_v2]<br>Show the field ONLY if:<br>[trae_v2] = '1'	Qual (is) medicamento (s) é (são) recomendado (s) para tratar a causa da Doença de Chagas?	checkbox, Required<br>1 medc_v2___1 Benzonidazol<br>2 medc_v2___2 Nifirtimox<br>3 medc_v2___3 Amiodarona<br>4 medc_v2___4 Propranolol<br>5 medc_v2___5 Não sei informar<br>9 medc_v2___9 Ignorado
	90	[cura_v2]	A doença de Chagas tem cura?	radio, Required<br>0 Sim<br>1 Não<br>2 Não sei informar<br>9 Ignorado

	91	[orien_v2]	Como o (a) Sr. (a) orientaria uma pessoa que encontrou um inseto "barbeiro/chupança" na sua residência?	radio, Required<br>1 Matar o inseto e eliminar o resíduo com limpeza profunda<br>2 Soltar o inseto em uma mata próxima<br>3 Colocar o inseto em um pote e entregar na Vigilância<br>4 Nada se deve fazer<br>5 Não sei informar<br>9 Ignorado
	92	[manip_v2]	Caso seja necessário manipular o "barbeiro/chupança", como esse processo deve ser feito?	radio, Required<br>1 Não precisa proteger as mãos para isso<br>2 Luvas ou proteção com saco plástico<br>3 Não devemos manipular barbeiro em nenhuma hipótese<br>4 Não sei informar<br>9 Ignorado
	93	[encam_v2]	Para qual (is) serviço (s) o (a) Sr (a) pode encaminhar o inseto barbeiro/chupança?	checkbox, Required<br>1 encam_v2___1 Posto de Informação de Triatomíneos (PIT)<br>2 encam_v2___2 Centro de Controle de Zoonoses (CCZ)<br>3 encam_v2___3 Secretaria Municipal de saúde (SMS)<br>4 encam_v2___4 Não sei informar<br>5 encam_v2___5 Ignorado
	94	[pproc_v2]	Caso uma pessoa seja picada pelo inseto barbeiro/chupança, o que o (a) Sr (a) orientaria?	radio, Required<br>1 Fazer testes sanguíneos e acompanhar a sorologia<br>2 Iniciar o tratamento para a doença imediatamente<br>3 Aguardar o aparecimento de sintomas<br>4 Não sei informar<br>9 Ignorado
	95	[info_v2]	Como o (a) Sr (a) teve acesso à informação sobre doença de Chagas?	radio, Required<br>1 Durante a graduação/ensino profissionalizante<br>2 Especialização<br>3 Mestrado<br>4 Doutorado<br>5 Curso de capacitação<br>6 Outro (a)<br>7 Nunca obtive informação alguma sobre Doença de Chagas<br>9 Ignorado
	96	[qidc_v2]<br>Show the field ONLY if:<br>[info_v2] = '6'	Qual a outra maneira pela qual obteve informação sobre Doença de Chagas?	text, Required
	97	[rcam_v2]	Section Header: *Bloco 4 - Práticas concernentes à Doença de Chagas desenvolvidas pelo setor saúde Nesse bloco, o (a) Sr (a) será perguntado sobre as Práticas desenvolvidas no setor saúde do município em que trabalha referentes à Doença da Chagas.*<br>A Secretaria Municipal de Saúde de Irecê realiza campanhas para a prevenção da Doença de Chagas?	radio, Required<br>0 Sim<br>1 Não<br>2 Não sei informar<br>9 Ignorado
	98	[ecam_v2]	A Secretaria Municipal de Saúde de Irecê estimula a realização de campanhas para a prevenção da Doença de Chagas na Unidade de Saúde da Família em que o (a) Sr. (a) trabalha?	radio, Required<br>0 Sim<br>1 Não<br>2 Não sei informar<br>9 Ignorado

	99	[prev_v2]	Existe algum programa de prevenção à Doença de Chagas na Unidade de Saúde da Família em que o (a) Sr. (a) trabalha?	radio, Required<br>0 Sim<br>1 Não<br>2 Não sei informar<br>9 Ignorado
	100	[ppeu_v2]<br>Show the field ONLY if:<br>[prev_v2] = '0'	Qual é o programa de prevenção para Doença de Chagas existente na USF?	text, Required
	101	[matdi_v2]	Na Unidade de Saúde da Família em que o (a) Sr (a) trabalha está disponível algum tipo de material para a orientação em casos suspeitos de Doença de Chagas, incluindo como proceder quando encontrar insetos perto ou dentro de casa?	radio, Required<br>1 Sim<br>2 Não<br>3 Não sei<br>9 Ignorado
	102	[bbic_v2]	A equipe em que o (a) Sr (a) trabalha realiza busca por barbeiros no interior das casas no território de abrangência da Unidade de Saúde da Família?	radio, Required<br>0 Sim<br>1 Não<br>2 Não sei<br>9 Ignorado
	103	[bbrc_v2]	A equipe em que o (a) Sr (a) trabalha realiza busca por barbeiros ao redor das casas no território de abrangência da Unidade de Saúde da Família?	radio, Required<br>0 Sim<br>1 Não<br>2 Não sei<br>9 Ignorado
	104	[pren_v2]	Na Unidade de Saúde da Família em que o (a) Sr (a) trabalha é realizada a pesquisa para a Doença de Chagas durante o atendimento pré-natal ?	radio, Required<br>0 Não<br>1 Sim<br>2 Não sei informar<br>9 Ignorado
	105	[rbtr_v2]	O (a) Sr (a) já recebeu de alguma pessoa um inseto "barbeiro/chupança" na Unidade de Saúde da Família onde trabalha?	radio, Required<br>0 Não<br>1 Sim<br>2 Não sei<br>9 Ignorado
	106	[slba_v2]	Na Unidade de Saúde da Família em que o (a) Sr (a) trabalha é realizada a coleta de sangue para a sorologia de Doença de Chagas quando é localizado pelo usuário ou pela equipe um "barbeiro/chupança"?	radio, Required<br>0 Sim<br>1 Não<br>2 Não sei<br>9 Ignorado
	107	[atpb_v2]	O (a) Sr (a) já atendeu na Unidade de Saúde da Família em que trabalha pessoas picadas pelo "barbeiro/chupança"?	radio, Required<br>0 Não<br>1 Não lembro<br>2 Não sei informar<br>3 Sim<br>9 Ignorado
	108	[sdch_v2]	O (a) Sr (a) já suspeitou de Doença de Chagas em alguma pessoa atendida na Unidade de Saúde da Família em que trabalha?	radio, Required<br>0 Não<br>1 Sim<br>2 Não sei informar<br>9 Ignorado

	109	[diag_v2]	Alguma pessoa foi diagnosticada com doença de Chagas na Unidade de Saúde da Família em que o (a) Sr. (a) trabalha?	radio, Required<br>0 Não<br>1 Sim<br>2 Não sei informar<br>9 Ignorado
	110	[atdc_v2]	Na Unidade de Saúde da Família em que o (a) Sr. (a) trabalha é realizado o atendimento às pessoas portadoras de Doença de Chagas?	radio, Required<br>0 Sim<br>1 Não<br>2 Não sei informar<br>9 Ignorado
	111	[mopc_v2]<br><br>Show the field ONLY if:<br>[atdc_v2] = '0'	Qual é o tipo de moradia das pessoas atendidas com Doença de Chagas na Unidade de Saúde da Família em que o (a) Sr (a) trabalha?	radio, Required<br>0 Tijolo/adobe<br>1 Madeira<br>2 Taipa revestida<br>3 Taipa não revestida<br>4 Material aproveitado<br>5 Não sei informar<br>9 Ignorado
	112	[baid_v2]<br><br>Show the field ONLY if:<br>[atdc_v2] = '0'	A equipe da Unidade de Saúde da Família em que o (a) Sr (a). trabalha procura por "barbeiros" dentro da casa das pessoas portadoras de Doença de Chagas aqui atendidas?	radio, Required<br>0 Sim<br>1 Não<br>2 Não sei informar<br>9 Ignorado
	113	[bapd_v2]<br><br>Show the field ONLY if:<br>[atdc_v2] = '0'	A equipe da Unidade de Saúde da Família em que o (a) Sr (a). trabalha procura por "barbeiros" ao redor da casa das pessoas portadoras de Doença de Chagas aqui atendidas?	radio, Required<br>0 Sim<br>1 Não<br>2 Não sei informar<br>9 Ignorado
	114	[questionrio_conhecimento_dos_trabalhadores_da_c7f8_complete]	Section Header: *Form Status*<br>Complete?	dropdown<br>0 Incomplete<br>1 Unverified<br>2 Complete
